# Pheochromocytoma, Fulminant Heart Failure, and a Phenylephrine Challenge. the Perioperative Management of Adrenalectomy in a Jehovah’s Witness Patient: a Case Report

**DOI:** 10.2478/jccm-2021-0038

**Published:** 2021-11-13

**Authors:** Sarah A. Bachman, Ryan S. Peterson, Peter S. Burrage, Leigh C Hickerson

**Affiliations:** 1 Dartmouth-Hitchcock Medical Center, Lebanon, NH, USA

**Keywords:** pheochromocytoma, systolic heart failure, stress cardiomyopathy, vasoplegia, bloodless medical and surgical procedures

## Abstract

Perioperative management of pheochromocytoma in the setting of catecholamine-induced heart failure requires careful consideration of hemodynamic optimization and possible mechanical circulatory support. A Jehovah’s Witness patient with catecholamine-induced acutely decompensated heart failure required dependable afterload reduction for a cardio-protective strategy. This was emphasized due to the relative contraindication to perioperative anticoagulation required for mechanical circulatory support. A phenylephrine challenge clearly demonstrated adequate alpha blockade after only 24 hours of phenoxybenzamine treatment. This resulted in advancement of the surgery date. This case also highlights management of beta blockade, volume and salt loading, autologous blood transfusion, and profound post-operative vasoplegia in the setting of cardiogenic shock. Careful attention to hemodynamic optimization and cardio-protective strategies ultimately resulted in positive outcome for this challenging clinical scenario.

## Introduction

The pathologic effects of a pheochromocytoma can mimic many cardiovascular syndromes. The associated catecholamine surges can precipitate cardiovascular events including aortic dissection, myocardial ischemia, cardiac arrhythmias, or Takotsubo’s cardiomyopathy [1, 2, 3]. Hypertension is the most common manifestation (56%), followed by palpitations (36%), angina (20%) and dyspnoea (16%) [2]. Non-specific ST-T changes and arrhythmias with troponin elevations are also common[4].

Surgical resection remains the mainstay of definitive pheochromocytoma management.

Preoperative management focuses on optimization of the hemodynamic parameters outlined in the Roizen criteria. These include aggressive treatment of hypertension, ventricular arrhythmias and myocardial ischemia [5]. Common pharmacologic approaches include alpha adrenergic receptor blockade titrated over a period of 7-14 days to produce orthostasis followed by beta blockade and volume loading [6]. Tyrosine hydroxylase inhibitors are viable alternatives to alpha adrenergic blockade and decrease endogenous catecholamine biosynthesis [7]. Magnesium directly antagonizes catecholamine receptors and calcium channel blockers are used for refractory hypertension, catecholamine-induced myocarditis, and vasospasm [8, 9].

Achieving Roizen criteria preoperatively may present a challenge when faced with a clinical scenario of acutely decompensated heart failure (ADHF) due to excessive catecholamine production. Cardiac output may be the predominant determinant of systemic blood pressure. Hypotension may result in poor end-organ perfusion. Low electrocardiogram voltage may obscure ST-T wave changes. Acutely blocking catecholamine receptors in the context of decompensated heart failure can precipitate worsening hemodynamic decompensation.[10] Orthostatic hypotension may be the only indicator of adequate sympathetic blockade in severe heart failure.[8] Similarly, typical preoperative salt and volume repletion may be poorly tolerated.

Hemodynamic goals for patients with pheochromocytoma and ADHF require careful adjustment (Table 1). Patients presenting in pulmonary edema from decreased cardiac output and elevated left-sided filling pressures may require mechanical circulatory support [2]. In the literature reviewed, the majority of pheochromocytoma-induced fulminant heart failure patients required left ventricular mechanical support including intra-aortic balloon pump, Impella device, or veno-arterial extracorporeal membrane oxygenation [2, 3, 9, 10, 11, 12].

**Table 1:** Perioperative goals for pheochromocytoma, stress cardiomyopathy, and acute decompensated heart failure [19]

	Pheochromocytoma	Stress Cardiomyopathy	ADHF
Preoperative	Alpha Blockade	Afterload reduction	Afterload reduction
Optimization	Antiarrhythmic	Antiarrhythmic	Antiarrhythmic/ICD
	Volume loading	Diuretics	Diuretics
	Beta Blockade	Beta blockade/ACEI (chronic	Beta blockade/ACEI (chronic
		stage/recovery) MCS	stage/recovery) MCS

Intraoperative	Afterload reduction Vol-	Afterload reduction	Maintain cardiac output
Hemodynamic Goals	ume resuscitation	Avoid volume overload	Avoid volume overload

Expected Recovery	10 days - 6 weeks	1-4 weeks	Chronic, progressive

The perioperative management of a 47-year-old male Jehovah’s Witness who presented in fulminant heart failure and was subsequently diagnosed with a large pheochromocytoma is described.

Informed consent was obtained prior to publication.

## Case Presentation

A 47 year old Jehovah’s Witness patient with a diagnosis of hypertension presented to the Emergency department at Concord Hospital, Concord, New Hampshire, United States of America (USA) with productive cough and dyspnoea. Upon presentation, a chest radiograph demonstrated bilateral consolidations. He was treated with a seven day course of ceftriaxone (Hospira, Lake Forest, Illinois, USA) and azithromycin (Pfizer, New York, New York, USA) followed by a fourteen day course of cefuroxime (Hikma, Berkeley Heights, New Jersey, USA) for multilobar pneumonia. Upon history, he had stopped taking lisinopril (Hikma, Berkeley Heights, New Jersey, United States of America) and amlodipine (Pfizer, New York, New York, USA) during a routine office visit one month prior due to medication non-compliance and normo-tension during the office visit.

Six weeks after his initial presentation, he presented again to the Emergency Department at Concord Hospital, Concord, New Hampshire, USA with productive cough and dyspnoea. His other symptoms included intermittent blurred vision, migraines, facial paraesthesia, anxiety, and a new onset diabetes.

His blood pressure upon presentation at the emergency department in Concord Hospital was recorded as a systolic pressure of 115 mmHg and diastolic pressure of 70 mmHg.

In the Emergency Department at Concord Hospital, he underwent an electrocardiogram, computerized tomography of the chest and abdomen, and magnetic resonance imaging of the brain. The electrocardiogram revealed sinus tachycardia with a ventricular rate of 130 beats per minute and left ventricular hypertrophy. Chest radiograph demonstrated interstitial oedema, bilateral pleural effusions and resolution of his previous consolidations. He was started on metoprolol (Hospira Inc, Lake Forest, Illinois, USA) and furosemide (Hospira Inc, Lake Forest, Illinois, USA). Magnetic resonance imaging of the brain displayed punctate haemorrhage in the right frontal lobe. The computerized tomography revealed bilateral pleural effusions, interstitial oedema, interval resolution of multifocal infiltrates and an incidental 9.2 cm left adrenal mass. He was admitted to Concord Hospital directly from the Concord Emergency Department.

On the day after admission to Concord Hospital, his echocardiogram demonstrated a left ventricular ejection fraction of 30%. Cardiac catheterization demonstrated no obstructive coronary pathology. Urine metanephrines collected between day one and two after admission to Concord Hospital demonstrated predominant epinephrine secretion with 2,313 mcg/day of epinephrine compared to 671 mcg/day of norepinephrine and 873 mcg/day of dopamine. Three days after admission, he was trialled on prazosin (Greenstone, Peapack, New Jersey, United States of America) which was immediately discontinued due to hypotension. He was then transferred to Dartmouth-Hitchcock Medical Center, Lebanon, New Hampshire, USA.

On the day of admission to Dartmouth-Hitchcock Medical Center, he was started on phenoxybenzamine (Par Pharmaceutical, Chestnut Ridge, New York, USA). On the day after admission, the patient developed orthostatic hypotension. Volume loading resulted in increased dyspnoea. Phenoxybenzamine was discontinued due to persistent hypotension. Chest radiography demonstrated cardiomegaly with pulmonary congestion and small, bilateral pleural effusions. Echocardiography demonstrated left ventricular dilation and reduction in left ventricular ejection fraction (LVEF) to 20%. Diuresis was initiated at this time and continued until five days after admission. This resulted in a fluid balance of net - 3 liters.

**Fig. 1:**
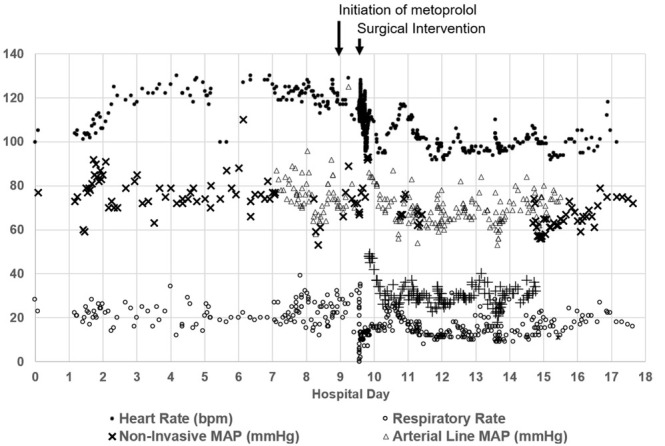
Hemodynamic fluctuations during hospitalization. Time markers for initiation of beta blockade and surgery are superimposed.

Five days after admission, he developed a sustained tachycardia and was moved to the intensive care unit, where an arterial line was placed. The phenoxybenzamine was resumed six days after admission and was increased to 20 mg twice daily over the next 24 hours without haemodynamic compromise (See figure 1).

Orthostatic hypotension was achieved 24 hours after resuming phenoxybenzamine. Gentle fluid loading was initiated on day six after admission. By day seven, his fluid balance for admission was neutral.

A multidisciplinary discussion between urology, anaesthesiology, heart failure cardiology, and endocrine teams focused on the benefit of urgent surgical resection versus delaying to ensure further time for alpha blockade.

Seven days after admission, an alpha agonist challenge was proposed to test the efficacy of alpha blockade. Graded doses of intravenous phenylephrine (CAPS, Leigh High Valley, Pennsylvania, USA) were administered with an initial bolus of 80 mcg followed by increasing doses administered at one minute intervals up to 800 mcg. No change occurred in systolic, diastolic or mean arterial pressures.

Eight days after admission, an esmolol infusion (WG Cricial Care LLC, Paramus, New Jersey, USA) was titrated over a 1 hour period from 10 mcg/kg/min to 200 mcg/kg/min with direct observation for mean arterial pressure less than 60 mm Hg, increased work of breathing, or change in mental status. The patient tolerated this with no new symptoms and he was started on metoprolol 12.5 mg every six hours (Hospira Inc, Lake Forest, Illinois, USA).

Surgery was scheduled nine days after admission. Mechanical circulatory support was not offered as a hemodynamic salvage technique during the case. This decision was made to avoid systemic anticoagulation, required for MCS in the setting of a high intraoperative haemorrhage risk, the large retroperitoneal surgical approach, and the patient’s refusal of all blood products.

Immediately prior to induction of anaesthesia, 8 mcg norepinephrine (Advanced Compounding Solutions, Woburn, Massachusetts, USA) and 1 unit vasopressin (Par Pharmaceutical, Chestnut Ridge, New York, USA) were administered with no haemodynamic effect. Five minutes later, an additional 2 units of vasopressin and 24 mcg norepinephrine were administered intravenously resulting in a raise in systolic blood pressure from 89 mmHg to 140 mmHg.

Intravenous induction was performed immediately after this titration with 0.75 mcg/kg of fentanyl (Hospira, Lake Forest, Illinois, USA), 2 mg/kg of propofol (Fresnius Kabi, Lake Zurich, Illinois, USA), 1.5 mg/ kg of lidocaine (Hikma Berkeley Heights, New Jersey, United States of America), and 1 mg/kg of rocuronium (Pfizer, New York, New York, USA). A transoesophageal echocardiography probe and pulmonary artery catheter were placed showing a baseline PAP in the low 30s mmHg. A transesophageal echocardiogram showed a left ventricular ejection fraction of 15% under anaesthesia and bilateral pleural effusions. Surgical incision and drainage of approximately 1600 ml of fluid from the left chest immediately improved respiratory mechanics initially compromised by atelectasis and supine positioning. Intraoperative tumour manipulation resulted in rapid increases in systolic blood pressure to 160s mmHg. This was associated with increases in PA diastolic pressures up to 50 mmHg and worsening contractility on TEE.

Thus, increases in blood pressure above mid 130s mmHg were treated with IV phentolamine and 1 gram bolus doses of magnesium sulphate (WG Critical Care, Paramus, New Jersey, USA).

Immediately following intra-operative ligation of venous return from the adrenal gland, hypotension ensued. Hemodynamic monitoring at this time demonstrated a cardiac index of approximately 2 L/min/ m^2^ with an epinephrine infusion (Par Pharmaceutical, Chestnut Ridge, New York, United States of America), consistent with a new distributive component on top of the existing cardiogenic shock picture. This was treated with rapidly escalating doses of norepinephrine (max infusion dose of 0.7 mcg/kg/min), vasopressin (max dose 0.08 units/min), epinephrine (0.07-0.2 mcg/kg/ min) with adequate blood pressures reached at doses of norepinephrine 50 mcg/min, vasopressin 0.08 U/min, and epinephrine at a rate of 10 mcg/min required for transport to the ICU. Refractory distributive shock was treated with hydrocortisone 250 mg IV (Pfizer, New York, New York, USA), 0.45 mg methylene blue IV infusion (American Regent, Columbus, Ohio, United States of America), 4 mg glucagon IV (Fresenius Kabi, Lake Zurich, Illinois, USA) and 400 mg calcium chloride IV (International Med Systems LTD, El Monte, California, USA).

The kidney and adrenal glands were excised en-bloc to reduce the potential blood loss that might result from excision of the nine cm tumour alone. A cell saver machine was available, but not required as intraoperative blood loss was estimated to be 300ml. Fluid boluses were judiciously given in 250 ml aliquots while carefully monitoring PA pressure data. Additional support with angiotensin II and thyroid hormone rescue therapies were considered, but ultimately not required. A right chest tube was placed by the thoracic surgical team prior to leaving the operating room and a further 1400 ml of pleural effusion was drained from that side and then left in place for intermittent drainage postoperatively.

On post-operative day zero, the patient was transported to the intensive care unit. Upon arrival, the pulmonary capillary wedge pressure was 37 mm Hg. This gradually decreased after repeated boluses of furosemide to 20 mmHg over the following 48 hours and vasopressors were weaned off over the next four days. His cardiac index during this time was consistently >2L/ min/m^2^. Post-operative creatinine peaked at 1.31 mg/ dL with BUN of 38 mg/dL and had returned to normal after post-operative day 3 as urine output was supported with diuresis. Post-operative haemoglobin nadired at 10.6 mg/dL from a preoperative value of 12.9 mg/dL and increased to 11.1 mg/dL prior to discharge. Lactate peaked at 2.6 mg/dL intraoperatively and had decreased to 1.6 mg/dL within hours post-operatively. The patient was uneventfully extubated on postoperative day four. Prior to discharge, his **left ventricular ejection fraction** had improved to 27%.

Three months after hospitalization, furosemide was discontinued. Six months after discharge from the hospital, he remained on metoprolol 12.5 mg (Hospira, Lake Forest, Illinois, USA). He no longer requires insulin and his haemoglobin A1c was 5.6%. His anxiety and migraines resolved post-operatively and he remains asymptomatic six months after discharge.

Tissue pathology confirmed pheochromocytoma with a PASS score 4-6. While genetic testing for MEN syndromes was recommended, it has not yet been performed.

At the time of writing this report, the patient remains followed-up by the heart failure clinic; he has an improved exercise capacity and is now able to hike and chop wood; however echocardiograms continues to show an ejection fraction of 30% with diffuse hypo kinesis. His proBNP has fallen from 3566 preoperatively to 172 on most recent labs.

## Discussion

This presentation of a patient in acute decompensated heart failure due to a catecholamine-induced cardiomyopathy in the context of a pheochromocytoma provided a challenge in both initial diagnosis and perioperative optimization. Catecholamine-induced ADHF from pheochromocytoma has been previously described in multiple case reports. A meta-analysis in 2017 reviewed 163 cases of pheochromocytoma-induced cardiomyopathy. The average ejection fraction was 22% and many classic symptoms of pheochromocytoma were not as common. For example, only 4% presented with hypertensive urgency which was also absent in this case report. Resection of the pheochromocytoma led to improvement in cardiomyopathy in 96% of patients [15]. The presence of ADHF may preclude typical perioperative management of pheochromocytoma. Titration of alpha and beta adrenergic blockade as well as salt and fluid loading all must be carefully considered if not altogether avoided.

In this case, a phenylephrine challenge was useful to demonstrate the adequacy of alpha blockade and thereby advanced his surgical date by several days leading to decreased time to definitive management. Confirming adequate alpha blockade was critical in this patient to reduce afterload in the context of severely reduced LVEF. In the literature, a phenylephrine challenge was previously described in an obstetric patient prior to adrenalectomy, however it was a gradual up-titration of phenylephrine following a typical course of alpha blockers for 14 days [13]. A phenylephrine challenge may prove more useful in testing alpha blockade efficacy prior to completion of a standard 14 day course.

The patient’s rapid and profound response to the phenoxybenzamine may have represented the down-regulation of alpha receptors due to excessively high circulating catecholamines. The extent of this patient’s alpha blockade was also highlighted by the profound vasoplegia that followed tumor removal requiring elevated doses of catecholamines and vasopressin to maintain mean arterial blood pressure. A pulmonary artery catheter was helpful in management of intra and postoperative volume optimization and allowed for monitoring of cardiac performance [7].

Blood conservation strategies such as en-bloc resection and autologous blood salvage were used to maximize oxygen carrying capacity. Pheochromocytoma is considered a relative contraindication for autologous blood salvage techniques because tumour catecholamines are not filtered in cell saver washing. The risks of expected hemodynamic changes with autologous blood transfusion were outweighed by the patient’s refusal of blood products [16, 17, 18]. Thankfully, his circulatory shock was not aggravated by intraoperative haemorrhage and autologous transfusion was not required.

Mechanical circulatory support is a useful adjunct intraoperatively, however it was relatively contraindicated due to the need for anticoagulation in the perioperative period in a patient who would not accept blood products. The intraoperative and post-operative risk of haemorrhage was high and therefore, mechanical circulatory support was not offered. Therefore, this case required additional careful attention to perioperative optimization. Afterload reduction was essential in this patient to protect the left ventricle during the afterload increases associated with surgical resection. Pheochromocytoma in the setting of acute decompensated heart failure, poses challenges to traditional perioperative management. While afterload reduction is a hemodynamic goal shared by the two pathologies, volume resuscitation and beta blockade should be carefully considered.

## Conclusion

There are conflicting perioperative management goals for pheochromocytoma and catecholamine-induced ADHF. A personalized strategy was necessary in this patient to weigh the appropriateness of initiating inotropic support vs. beta blockade, diuresis vs. volume loading and alpha blockade in the setting of cardiogenic shock. MCS would have been a useful tool to support cardiac output. However, it was contraindicated in this patient due to refusal of transfusion. Afterload reduction was critical, but alpha blockers could not be up titrated due to hypotension. Therefore, a phenylephrine challenge was useful to ensure alpha blockade despite low doses of medication and decreased the time to surgical resection. Highly collaborative multidisciplinary perioperative planning was key to successful pheochromocytoma resection and subsequent hospital course in this complex patient.
